# Evolution and success of holmium laser enucleation of the prostate

**DOI:** 10.4103/0970-1591.70582

**Published:** 2010

**Authors:** Amy E. Krambeck

**Affiliations:** Mayo Clinic, Rochester, Minnesota, USA

**Keywords:** Benign prostatic hyperplasia, holmium laser, prostate enucleation

## Abstract

**Aims:**

The purpose of this article is to review the development of instruments, current technique, and expected outcomes for holmium laser enucleation of the prostate (HoLEP).

**Materials and Methods:**

A review of published, peer-reviewed articles focusing on HoLEP was performed using the MEDLINE database.

**Results:**

Historically, the gold-standard management for symptomatic obstructing benign prostatic hyperplasia (BPH) has been transurethral resection of the prostate (TURP). With the development of new laser technology minimally invasive surgical procedures have been introduced in an attempt to decrease the morbidity experienced with standard TURP. Laser treatment of BPH has evolved from coagulation to complete adenoma enucleation. The holmium laser was initially utilized for prostate ablation and soon evolved into holmium laser tissue resection, but was limited by difficulties with extracting the prostate tissue from the bladder. With the development of a compatible tissue morcellator whole prostate lobes could be enucleated similar to an open prostate enucleation and the HoLEP procedure was developed. Currently HoLEP is the only procedure to demonstrate superior outcomes to TURP on urodynamic studies and long-term studies demonstrate its durability up to 7 years post procedure. Changes in enucleation technique have also increased the efficiency of the HoLEP procedure, such that any sized prostate can be treated.

**Conclusions:**

HoLEP is a safe and effective surgical treatment for symptomatic BPH, dependent on a high powered laser and morcellation system. The procedure continues to gain acceptance due to excellent short and long-term results, its wide application, and further simplification of technique.

## INTRODUCTION

Transurethral resection of the prostate (TURP) is still considered the gold standard treatment for benign prostatic hyperplasia (BPH). However, its current use is limited to small and medium-sized prostates due to an overall morbidity rate of 15-20%[1] and blood transfusion rate ranging from 5% to 11%.[2] Patients currently undergoing treatment for BPH are progressively older with more comorbidities; thus, there is an increased need for more minimally invasive procedures in the current treatment era. In an attempt to limit the morbidity associated with standard TURP several laser therapies have been introduced for the treatment of BPH, including neodymium:yttrium aluminium garnet (Nd:YAG), the holmium:YAG, and the potassium-titanyl-phosphate (KTP) lasers.[3] These lasers have been used to coagulate, vaporize and cut prostatic tissue overgrowth using a variety of techniques. The holmium laser has been further developed to allow for actual prostatic lobe enucleation with subsequent tissue removal.

Holmium laser enucleation of the prostate (HoLEP) has emerged as an effective transurethral treatment option for patients suffering from symptomatic BPH of any size.[4] A multitude of publications have supported the safety and efficacy of HoLEP for small and large gland BPH,[4–20] even in the presence of bleeding diatheses and anticoagulation.[17] HoLEP has been found to be as effective as TURP[7–11,19] and open suprapubic prostatectomy[5,7,16] for the treatment of obstructive BPH, with the benefit of less morbidity. Long-term studies of patients undergoing HoLEP demonstrate sustained relief from BPH symptoms from 4 to 7 years postoperative, with very low retreatment rates, ranging from 0 to 4%.[12,16,18,21]

The efficacy of HoLEP lies in its excellent tissue debulking capabilities. Large case series have shown that HoLEP produces a prostate volume and prostate-specific antigen reduction of 60-90%.[6,13–15,18] Another benefit of HoLEP is the potential to be performed as an outpatient procedure with catheter removal within 24 hours of surgery. When compared to contemporary ablative procedures, HoLEP has the advantage of actual tissue removal for pathologic specimen examination, greater prostate volume reduction, and durable long-term results, while maintaining low morbidity.[22]

Since HoLEP is a laser based procedure it is performed using normal saline irrigant, thus eliminating the risk of dilutional hyponatremia, also known as TUR syndrome. Furthermore, since the laser can perform pin-point coagulation of bleeding vessels as they enter the capsule of the prostate the need for blood transfusion has nearly been eliminated in patients without bleeding diathesis. Evidence demonstrates the feasibility of radical prostatectomy after HoLEP; the concomitant treatment of bladder, ureteral and renal stones at time of HoLEP; and the limited impact of HoLEP on erectile function.[23] Investigators have reported that once the initial investment for the laser is factored out HoLEP is more cost-effective compared with TURP and open prostatectomy due to a shorter length of hospitalization and decreased need for ancillary interventions (i.e. blood transfusion, and continuous bladder irrigation).[19,24] The goal of this report is to describe how the HoLEP technique evolved, the current available equipment, and expected outcomes using current techniques.

## DEVELOPMENT OF HoLEP

The HoLEP procedure has evolved from a combination procedure with neodymium:yttrium-aluminum-garnet (Nd:YAG), to an ablative procedure, to an excisional technique involving resection of small fragments, to finally anatomic enucleation of whole prostate lobes. Holmium laser prostate surgery was pioneered in New Zealand and much of what is described was introduced by Gilling and colleagues.

Initially the holmium laser was used with the Nd:YAG laser to perform prostate ablation in a procedure termed “combination endoscopic laser ablation of the prostate”.[25] The Nd:YAG laser is a solid-state laser with a very low absorption coefficient so that it penetrates tissue deeply between 4 and 18 mm. The end result is coagulative necrosis and delayed sloughing of prostate tissue; however, the hemostatic effect is excellent.[3] The low energy holmium laser was combined with the Nd:YAG in an attempt to reduce the short-term sequelae of tissue sloughing, which resulted in prolonged catheterization and delayed symptomatic improvement.

The first pure holmium laser ablation of the prostate (HoLAP) was reported in 1994 using a 60 Watt holmium laser.[26] As originally described, a side fire laser fiber is used to ablation the surface of the prostate in a near contact mode. The tissue is instantly vaporized and the procedure is continued until a TURP like cavity remains. Unlike other laser sources with greater depth of penetration, there is minimal “unseen” tissue damage when HoLAP is performed. Although the HoLAP procedure was relatively simple to learn it was time consuming with lower wattage lasers. Until recently, the holmium laser vaporization technique had largely been abandoned, since it was felt to be inefficient and generated no surgical specimen. However, due to recent reintroduction of the HoLAP by industry as a possible bridge technique to HoLEP the procedure has re-emerged as a more popular treatment alternative. Furthermore, now that the 60 Watt laser has been replaced with the 100 Watt laser, ablation of larger glands is possible in a more efficient manner.

In an attempt to increase the efficacy and efficiency of holmium laser prostate surgery the holmium laser resection of the prostate (HoLRP) technique was developed.[26] This procedure is performed with either an end or side fire laser fiber and takes advantage of the precise incisional qualities of the holmium wavelength.[27–29] In HoLRP, the entire prostatic adenoma is removed in a piecemeal fashion by cutting the prostate lobes into pieces small enough for evacuation through the resectoscope sheath. The end result is that the adenoma is completely resected down to the prostatic capsule.[30] A major benefit of the HoLRP technique over ablation is that prostate tissue is available for pathology evaluation. Approximately one third of the resected weight of tissue is retrieved, with the remainder vaporized;[10] however, the quality of the prostate tissue is somewhat inferior to that obtained at time of TURP due to thermal artifacts.[31] When compared to laser ablation of the prostate the HoLRP technique was found to have a shorter catheterization time, lower recatheterization and reoperation rates, and less irritable lower urinary tract symptoms.[32] When compared with TURP, the HoLRP procedure had similar urodynamic, continence, and potency outcomes.[33] One major disadvantage, which prevented widespread acceptance of the HoLRP technique, was longer operative times. The incision of the prostate lobes into fragments small enough for evacuation prolonged the procedure substantially.

It was not until 1996 when a viable mechanical morcellator was developed[34] to remove prostatic tissue fragments that complete lobe enucleation could be performed to give rise to the HoLEP procedure. In the HoLEP procedure the holmium laser fiber and laser scope move in the same plane as the surgeon’s index finger would in an open prostatectomy to shell out the prostatic adenoma. Bleeding vessels are coagulated as the lobe is enucleated by moving the laser away from the capsular tissue. The enucleated lobe is then deposited in the bladder where it is removed using a tissue morcellator. The tissue morcellator allowed for significantly shorter operative times compared to HoLRP[34,35] making the procedure more attractive. The hemostatic properties of the holmium laser and the use of normal saline irrigant enabled HoLEP to be performed on prostates of all sizes; resulting in very low transfusion rates and eliminating the risk of resection related hyponatremia.

## CURRENT AVAILABLE EQUIPMENT

**Figure 1:**
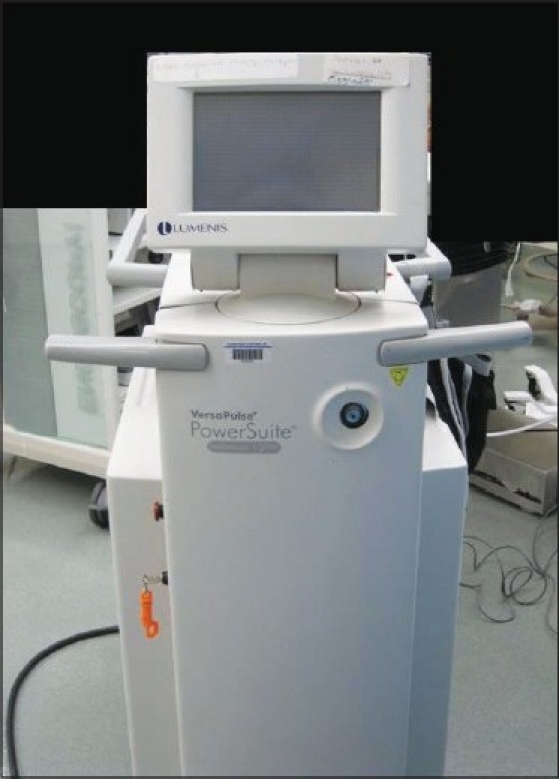
The 100 watt Versapulse holmium laser used to perform HoLEP

The holmium laser is a pulsed solid-state laser with a wavelength of 2140 nm. Unlike other available laser systems, the holmium laser is a contact laser with a depth of penetration in prostatic tissue of only 0.4 mm. The laser is highly absorbed by water (absorption peak of water: 1.940 nm), which makes up 60-70% of the prostate.[23] This water absorption produces an energy density that heats the prostatic tissue to greater than 100 degrees Celsius.[3] With such high heats created, the tissue is vaporized without deep coagulation for a “what you see is what you get” effect, eliminating delayed tissue sloughing. The holmium laser produces very little char effect, which allows the laser to precisely cut and dissection tissue it is in direct contact with without obscuring surgical planes. When the laser is not in direct contact with the tissue it can dissipate heat causing coagulation of vessels to a depth of 2 to 3 mm.[3] The holmium laser is a multipurpose laser and can be used not only for tissue cutting (as in the treatment of urinary strictures) and coagulation (treatment of urothelial tumors) but also for fracturing of stones.[36–38] To perform HoLEP in an efficient manner a high powered laser is necessary and in general the 100 watt Versapulse holmium laser (Lumenis, Santa Clara, CA) is used [Figure 1].

**Figure 2:**
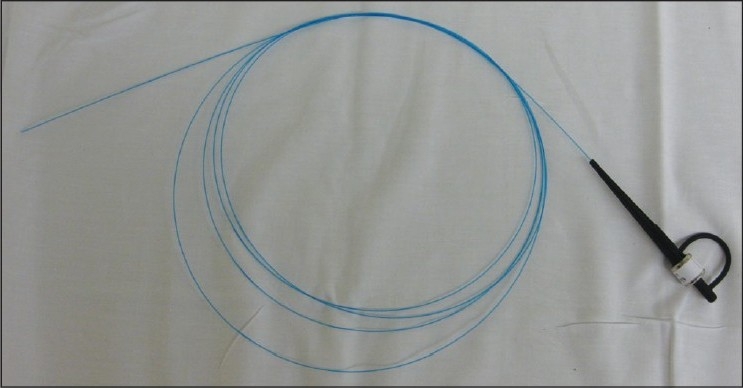
The 550 μm quartz laser fi ber used to perform HoLEP

The holmium laser energy can be transmitted along flexible quartz fibers of varying diameters, ranging from 200 to 100 μm. The ability to use multiple sized fibers allows the holmium laser to be used with not only a cystoscope, but also rigid and flexible ureteroscopes. In general, a larger laser fiber is necessary to perform HoLEP, and the 550 μm end-fire fiber is generally preferred [Figure 2]. Several different companies offer both disposable and reusable quartz laser fibers. The ability to sterilize and reuse the laser fibers up to 20-30 uses gives HoLEP a theoretical economical advantage over other prostate laser treatments.[23] When performing HoLEP the laser fiber is routinely stripped of its protective cladding, over several inches, and then placed through a 6 Fr stabilizing catheter (Cook, Spencer, IN). The catheter is secured in place with a Luer-Lok injection port (Baxter, Deerfield, IL).

**Figure 3:**
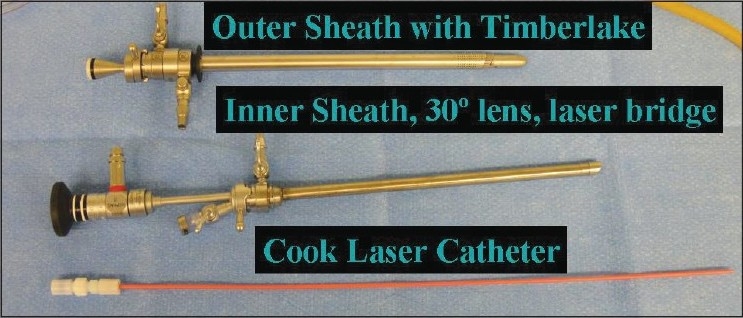
The disassembled laser scope and protective laser catheter. The device shown is the Storz 28 Fr set consisting of a 28 Fr outer sheath, inner sheath with stabilizing ring, and 30 degree telescope lens. The laser catheter fi ts through the working element of the scope and is held in place by the stabilizing ring.

Two different companies provide laser scopes that can be used to perform HoLEP. Olympus (Hamburg, Germany) has a 27 Fr continuous flow resectoscope with a modified inner sheath that incorporates a laser fiber channel and bridge [Figure 3]. Storz (Tuttlingen, Germany) manufactures two different sized continuous flow laser scopes to perform HoLEP; a 26 Fr instrument with a dedicated inner sheath and stabilizing guide and a 28 Fr instrument with a dedicated inner sheath and stabilizing ring. Regardless of laser scope used to perform HoLEP a 30 degree lens is necessary to adequately visualize the prostate and laser fiber. Due to the extreme hand movements necessary to perform HoLEP an endoscopic camera with a swivel base is necessary, as direct use of the eyepiece is neither feasible nor safe. High definition video systems such as those provided by Stryker (Kalamazoo, MI) and Olympus (Hamburg, Germany) make visualization of the plane between capsule and adenoma much easier, but are not necessary to perform the procedure. Since HoLEP is a laser based therapy normal saline irrigation is used in all cases.

**Figure 4:**
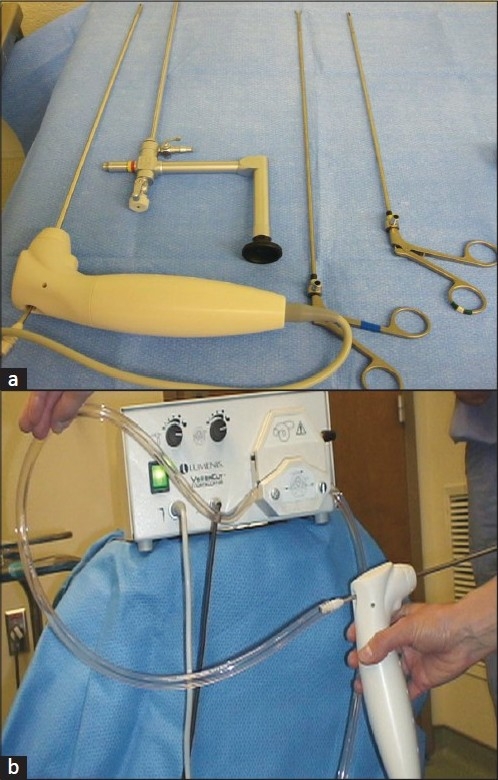
(a) The long nephroscope shown here has a 5 mm working channel which permits passage of the morcellator as well as grasping forceps. The grasping forceps can be used to remove small fragments rather than morcellating. (b) The Lumenis Versacut morcellator has a pump suction device which allows for simultaneous removal of the prostate tissue at time of morcellation

Once enucleation of the prostate has been completed the tissue must be removed using a tissue morcellator. To introduce the tissue morcellator the inner working elements of the laser scope are removed and replaced with a modified offset long 26 Fr nephroscope with a 5 mm working channel (Olympus or Storz). The tissue morcellator is then introduced through the 5 mm working channel [Figure 4a]. The Versacut morcellator (Lumenis) consists of a handpiece with reciprocating blades, controller box with suction pump and is operated solely by a foot pedal [Figure 4b]. Partial depression of the foot pedal produces suction only and complete depression allows for movement of the morcellator blades with suction. Due to the intense suction produced by the morcellator it is important to have 2 water inflows through the nephroscope to keep the bladder distended, preventing inadvertent damage by the morcellator. The Richard Wolf company (Vernon Hills, IL) has also developed a complete laser resectoscope which includes a morce-scope (i.e. a morcellator incorporated into a scope), which could also be used for tissue extraction. After all tissue is removed a standard urethral catheter is placed for at least 6 hours or until hematuria has decreased to an acceptable amount.

## CHANGES IN TECHNIQUE

Although the principals of enucleation remain the same, to develop the plane between the prostatic adenoma and the capsule just like open suprapubic prostatectomy, several modifications have been made to the HoLEP technique to improve efficacy. Previous studies have demonstrated that approximately 0.52 grams of tissue can be removed per minute, which includes morcellation, and that tissue removal efficiency increases with increasing gland size.[39] The phenomenon of increasing efficiency with increasing gland size is unique to HoLEP and has not previously been reported for other endoscopic prostate procedures. In fact, the risk of adverse effects such as bleeding and TUR syndrome increase with the TURP procedure proportionally as gland size increases.[1] Since the time to complete tissue morcellation relies almost solely on the morcellator equipment, urologists performing HoLEP have focused on alterations in the technique of enucleation to improve surgical efficiency.

Dusing and colleagues have presented several alterations in the enucleation technique at the annual American Urological Association meeting, which statistically increased HoLEP efficiency.[24,40] The authors site five changes to the HoLEP technique. The first alteration was to make a solitary posterior groove and incorporate the median lobe dissection into one of the lateral lobes. They state a single posterior groove is technically feasible for all but the most massive of median lobes. The second change was the initiation of the later lobe enucleation at the apex lateral to the verumontanum where the plane between the adenoma and capsule is prominent, as opposed to 12 o’clock anteriorly. The third change involves extending the lateral lobe dissection across the anterior plane over to the opposite side of the prostate from the apex to the bladder neck. The creation of the anterior potential space provides an easily identifiable target when the anterior commissure is divided to separate the right from the left lateral lobes of the prostate. The fourth modification is called the encircle technique, which lifts the prostate away from the sphincter so the apical mucosal strip can be quickly divided. Once the anterior lateral and posterior aspects of the distal prostate have been enucleated, the scope is rotated around the prostate starting anteriorly moving posteriorly and then pulled distally allowing the mucosal strip to present itself on tension for easy division away from the sphincter muscle. Finally, the fifth modification is specific for very large glands, where it can be difficult to deposit the enucleated adenoma into the bladder. They suggest that in these challenging cases the tissue should be morcellated in the prostatic fossa down to a small stalk which can then be manipulated to allow for completion of the enucleation.

## EXPECTED RESULTS

Since the HoLEP procedure is a complete debulking of the prostate it is of little surprise that the procedure produces durable long-term outcomes. Naspro and colleagues recently reviewed the literature for HoLEP and reported durable results at a mean follow-up of 43.5 months. They found a mean post procedure Qmax of 21.9 ml/sec and mean reoperative rate of 4.3% (range 0-14.1%).[23] The authors also noted a significant mean decrease in serum PSA levels from baseline (mean 63 ng/dl to 1.63 ng/dl, postoperatively) and transrectal ultrasound prostate volume (mean: from 68 ml to 27.2 ml, postoperatively). At longest follow-up the overall re-intervention rate was low at 0 to 5.4%.

Recently the group from Methodist Hospital in Indianapolis, Indiana evaluated their experience with over 1000 HoLEP procedures performed.[21] The mean preoperative transrectal ultrasound prostate volume was 99.3 grams (range 9 to 391), American Urological Association (AUA) symptom score 20.3 (1 to 35) and Qmax 8.4 cc/sec (1.1 to 39.3). Overall complication rates were low, occurring in only 2.3% of the cohort. Mean follow-up was 287 days, ranging from 6 days to 10 years. At most recent follow-up the mean AUA symptom score was 5.3, and Qmax was 22.7 cc/sec. Only 3 (0.3%) of patients were in urinary retention and the authors site that all three patients had findings consistent with an atonic bladder, not obstruction. Only one patient underwent a second HoLEP procedure for bleeding prostatic regrowth, not obstruction. Urethral stricture and bladder neck contractures occurred in less than 2% of the cohort.

Immediately postoperatively patients undergoing HoLEP can experience mild to moderate storage symptoms in the form of urgency and even urge incontinence. By one month postoperatively the symptoms are present in approximately 30% of patients and by 3 months only 10%.[23] The symptoms respond well to anticholinergic therapies and pelvic floor exercises, and in general are self limiting. The recent series of over 1000 HoLEP procedures reports a less than 5% overall incontinence rate at long-term.[21]

HoLEP appears to have limited impact on sexual function, similar to TURP and open suprapubic prostatectomy.[23] No difference in IIEF erectile function domain scores has been observed pre to 2 years postoperatively. However, patients should be counseled on the development of retrograde ejaculation, which has been noted in over 75% of patients followed over 6 years.[12]

## CONCLUSIONS

Many different surgical interventions exist for the treatment of BPH. With technologic advancements and development of technique HoLEP has emerged as an ideal treatment option. Short-term results demonstrate minimal morbidity making it safe for patients with significant comorbidities and for prostates of any size. Long-term results demonstrate durable de-obstruction with very few patients needing subsequent procedures. HoLEP relies on a high powered holmium laser, a compatible tissue morcellator and advanced surgical techniques for its success.
